# Alleviation of salt stress in *Glycyrrhiza uralensis* by lanthanum nitrate: a predictive modeling approach

**DOI:** 10.3389/fpls.2025.1659108

**Published:** 2025-09-22

**Authors:** Min Shang, Tingting Jia, Miao Ma

**Affiliations:** ^1^ College of Life Sciences, Shihezi University, Shihezi, Xinjiang, China; ^2^ Xinjiang Production and Construction Corps Key Laboratory of Oasis Town and Mountain-basin System Ecology, Shihezi, Xinjiang, China; ^3^ Key Laboratory of Xinjiang Phytomedicine Resource Utilization, Ministry of Education, Xinjiang, China

**Keywords:** Glycyrrhiza uralensis, lanthanum nitrate, root yield, medicinal quality, physiological mechanism, regression prediction

## Abstract

Seedling growth of *Glycyrrhiza uralensis* is severely inhibited by salt stress, limiting its cultivation in saline-alkali soils. This study aimed to evaluate the mitigative effects of lanthanum nitrate (La(NO_3_)_3_) and develop a predictive model for the salt stress response. The results showed that the photosynthesis, anti-oxidative stress, growth and relative content of pharmacological active components of *G.uralensis* were significantly inhibited under salt stress. Under 0.75 mM exogenous La(NO_3_)_3_ treatment, licorice under salt stress showed photosynthetic compensation recovery, activation of antioxidant defense, synergistic improvement of biomass and medicinal quality. At the same time, this study innovatively constructs the NRBO-LSSVM-ABKDE coupling prediction model, and its prediction accuracy is significantly better than the traditional algorithm, and the mitigation effect of La(NO_3_)_3_ on *G.uralensis* was successfully verified. These findings not only confirm the efficacy of La(NO_3_)_3_ in alleviating salt stress in *G. uralensis* but also provide a powerful predictive tool for assessing plant stress responses, offering a new strategy for sustainable agriculture in salt-affected areas.

## 1 Introduction

Soil salinization is one of the serious threats to sustainable development of global agriculture (Chen et al., 2024b; Gao et al., 2025), it is hindering the increase of crop yields in countries around the world, and severe salt stress can even lead to crop death. However, the hazard saline soil might become arable land for cultivating salt-tolerant economic plants (Jiashen et al., 2023). So strategic selection and cultivation of salt-tolerant crops with significant economic value on such lands present an effective and expeditious approach to augment the productivity of soil rich in salts (Arora et al., 2024).

*Glycyrrhiza uralensis* Fisch. is a medicinal and edible plant belonging to the Leguminosae, its dry root serving as a traditional medicinal material, which exhibits various functions including anti-inflammatory, antibacterial, antitumor, relieve a cough, hepatoprotective, immune-regulating, and antioxidant properties (Kwon et al., 2020), the glycyrrhizin and glycyrrhetinic acid in its root are 50 and 250 times sweeter than sucrose, respectively (Shadma et al., 2021). Therefore, its root and root extracts were widely used in the production of pharmaceuticals and foods. However, overexploitation led to a critical reduction in no matter its wild population number or population size, making cultivated licorice in the plantation to be as a substitute to the wild herb (Yang et al., 2022). Although adult *G. uralensis* had a higher tolerance to soil salinity stress (Hua et al., 2022), their seedlings exhibited weaker salt tolerance and often suffered from root rot and high mortality steming from the salt stress (Fan et al., 2024), significantly reducing their planting potential in saline lands. Up to now, extensive studies have shown that the application of exogenous substances was one of the most effective strategies to improve the seedlings’ salt tolerance (Fan et al., 2024; Ming et al., 2023; Xinhui et al., 2018). Although stress environments might promote the concentration of some medicinal components in the root of *G. uralensis*, they significantly inhibited the root growth and reduced yield of the medicinal organ, leading to a marked decrease in the content of the components (Xinping et al., 2024). To this end, we hope to find an innovative method that can not only effectively increase the concentration of the functional compounds in the licorice’s root under salt stress, but also significantly promote its root biomass, thereby increasing both the yield and the quality of the medicinal and edible organ.

Rare-earth elements, a group of non-essential but ubiquitous elements in organisms, have emerged as promising candidates in agricultural applications (Kaur et al., 2024, Zi et al., 2020). Lanthanum is one of the most abundant rare-earth elements in soil, which is widely used in agricultural production in the form of fertilizer ingredient (Duarte et al., 2018). Critically, lanthanum has demonstrated superior efficacy in enhancing plant salt tolerance compared to several other rare-earth elements in preliminary screenings and existing literature, particularly in modulating ionic homeostasis and antioxidant defense systems (Caigao et al., 2023; Tong et al., 2024). Appropriate concentration of lanthanum significantly improved crop growth and enhanced their tolerance to stress environments. However, the efficacy of lanthanum was contingent upon the specific plant species and the concentration at which it was administered.

Traditional experimental methods mostly use single factor or orthogonal design to optimize the processing of *G.uralensis*. However, due to the nonlinear and multivariable coupling characteristics of its physiological reaction process, it is difficult to fully reveal the interaction between various factors by relying solely on empirical design and linear analysis, and it is impossible to achieve accurate prediction and optimization of complex processing parameters. While previous studies have explored various strategies, including rare-earth elements, for mitigating salt stress in *G. uralensis*, a significant research gap remains: none have integrated advanced predictive modeling to systematically optimize the application parameters (specifically concentration) of lanthanum nitrate for maximizing salt tolerance. With the rapid development of artificial intelligence and machine learning technology, support vector machine (SVM) has been widely used in predictive modeling tasks in agriculture, environment, biomedicine and other fields due to its good generalization ability and advantages in small sample and nonlinear modeling (Sangar and Rajasekar, 2025; Tirandasu and Yalla, 2025; Yang et al., 2025). However, the standard SVM model still has some limitations in dealing with parameter tuning and high-dimensional complex data. To address this gap and achieve precise optimization of lanthanum nitrate application under salt stress, this study introduces a novel multivariate coupling prediction model. We constructed a multivariate response model around key physiological indicators, and innovatively introduced the Critic objective weighting method to construct a comprehensive index system suitable for the salt stress response test of *G. uralensis*, to ensure the rationality and discrimination of the weight of each index in the system (Liu et al., 2023). On this basis, this paper proposes, for the first time in this context, a hybrid model integrating Newton-Raphson Based Optimization (NRBO) (Ravichandran et al., 2024), Least Squares Support Vector Machine (LSSVM) (Waghmode et al., 2025) and Adaptive Bandwidth Kernel Density Estimation (ABKDE) (Zougab et al., 2014). This newly developed NRBO-LSSVM-ABKDE model is designed to accurately simulate and predict the optimal lanthanum nitrate concentration for enhancing salt tolerance in *G. uralensis* seedlings, representing a significant methodological advancement.

In addition, in order to verify the advantages of the proposed model in prediction accuracy and stability, this paper introduces three comparative models constructed by mainstream population optimization strategies, including particle swarm optimization algorithm-least squares support vector machine (PSO-LSSVM) (Liu et al., 2024b), northern eagle optimization algorithm-least squares support vector machine (NGO-LSSVM) (Liu et al., 2024a) and crown porcupine optimization algorithm-least squares support vector machine (CPO-LSSVM) (Yan et al., 2025). Through comprehensive performance comparison and analysis, the applicability of different optimization algorithms in physiological response modeling is systematically evaluated, so as to provide methodological reference and technical support for subsequent complex plant stress response modeling.

In summary, the effects of La(NO_3_)_3_ on photosynthetic gas exchange capacity, antioxidant enzyme activity, root growth, root biomass and accumulation of secondary metabolites of *G.uralensis* seedlings under NaCl treatment were studied, and the optimal amount of lanthanum nitrate to improve the salt tolerance of *G.uralensis* was determined. The prediction method of stress degree based on non-destructive testing can be used to guide the rapid implementation of remedial measures for artificial planting of licorice, so as to reduce the loss to the minimum.

## 2 Materials and methods

### 2.1 Materials

The seeds of *G. uralensis* used in this experiment were provided by Institute of Licorice in Shihezi University, and were identified by Prof. Miao Ma.

### 2.2 Plant growth conditions

**Figure 1:**
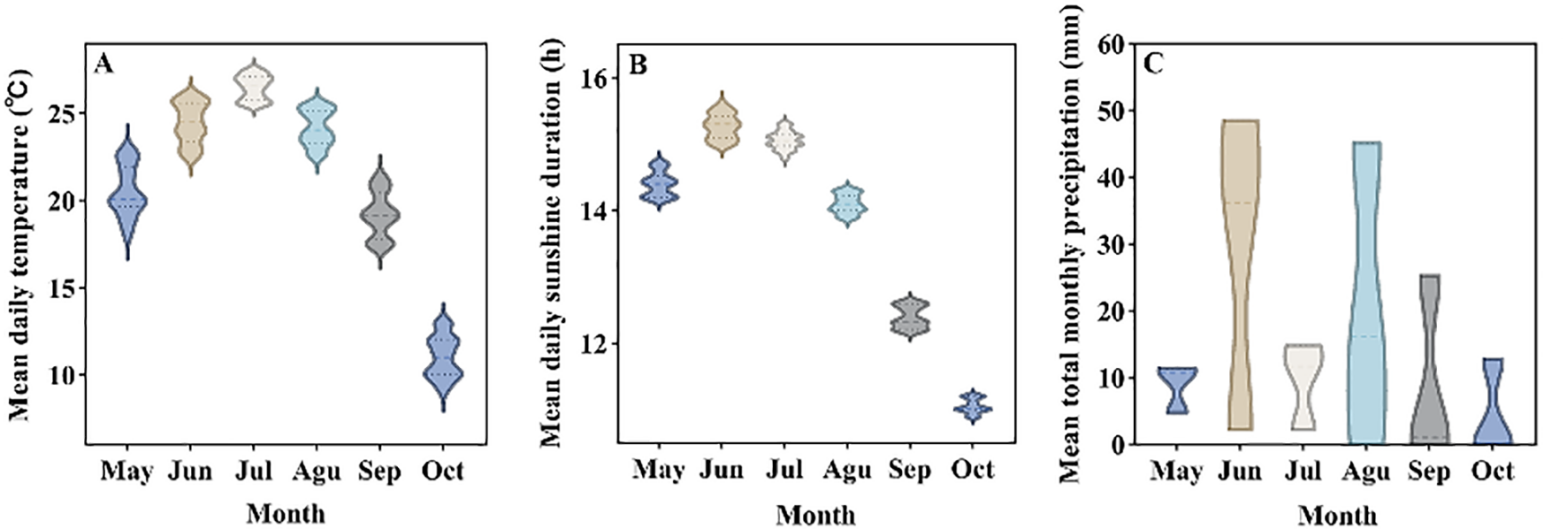
Climatic conditions of the experimental site. **(A)** Average daily temperature, **(B)** Average sunshine duration, **(C)** Average total monthly precipitation.

The experimental site was on the campus of Shihezi University (44°30′80′′N, 86°05′66′′E). The primary climate type in Shihezi was temperate continental climate, with long sunshine time in summer, dry and hot, and cold in winter, with an average altitude of 450 m. **Figure 1** shows the variation in average daily temperature (A), average sunshine duration (B) and average monthly precipitation (C) in the experimental site from May to October 2021. The temperature, sunshine duration and visible solar hours data were collected from the China Meteorological Science Data Network (https://data.cma.cn/).

### 2.3 Experimental design

Thirty plastic pots (25 cm in diameter and 25 cm in height) was selected, there were a total of 6 treatments, each treatment repeated 5 pots, and sandy loam (sand: soil mixed ratio of 3:7) was used as the cultivation substrate, 6 seeds of *G. uralensis* were evenly sown in each pot on May 15, 2021, and the sowing depth was 1 cm. All pots were co-located within a single homogeneous experimental plot (10 m × 15 m) to ensure identical macroclimatic exposure. Natural temperature/precipitation regimes were uniformly experienced across treatments (confirmed by hourly meteorological station data 50 m from plot). Critical climatic variables (temperature, precipitation) are quantified in **Figure 1**.

As the third leaf emerged, each plastic pot was fertilized with urea (N ≥ 46%) 14.99 g·m^-2^, calcium superphosphate (P_2_O_5_ ≥ 46%) 23.99 g·m^-2^, potassium sulfate (K_2_O ≥ 50%) 10.49 g·m^-2^ mixed fertilization, the fertilizer application was divided into 5 times, once every 20 days. The plastic pots were arranged in a random block design, and the positions were randomly changed once a week. During the whole experiment, plants were watered daily, and 60% of the relative water holding capacity was ensured by the weighing method.

**Figure 2:**
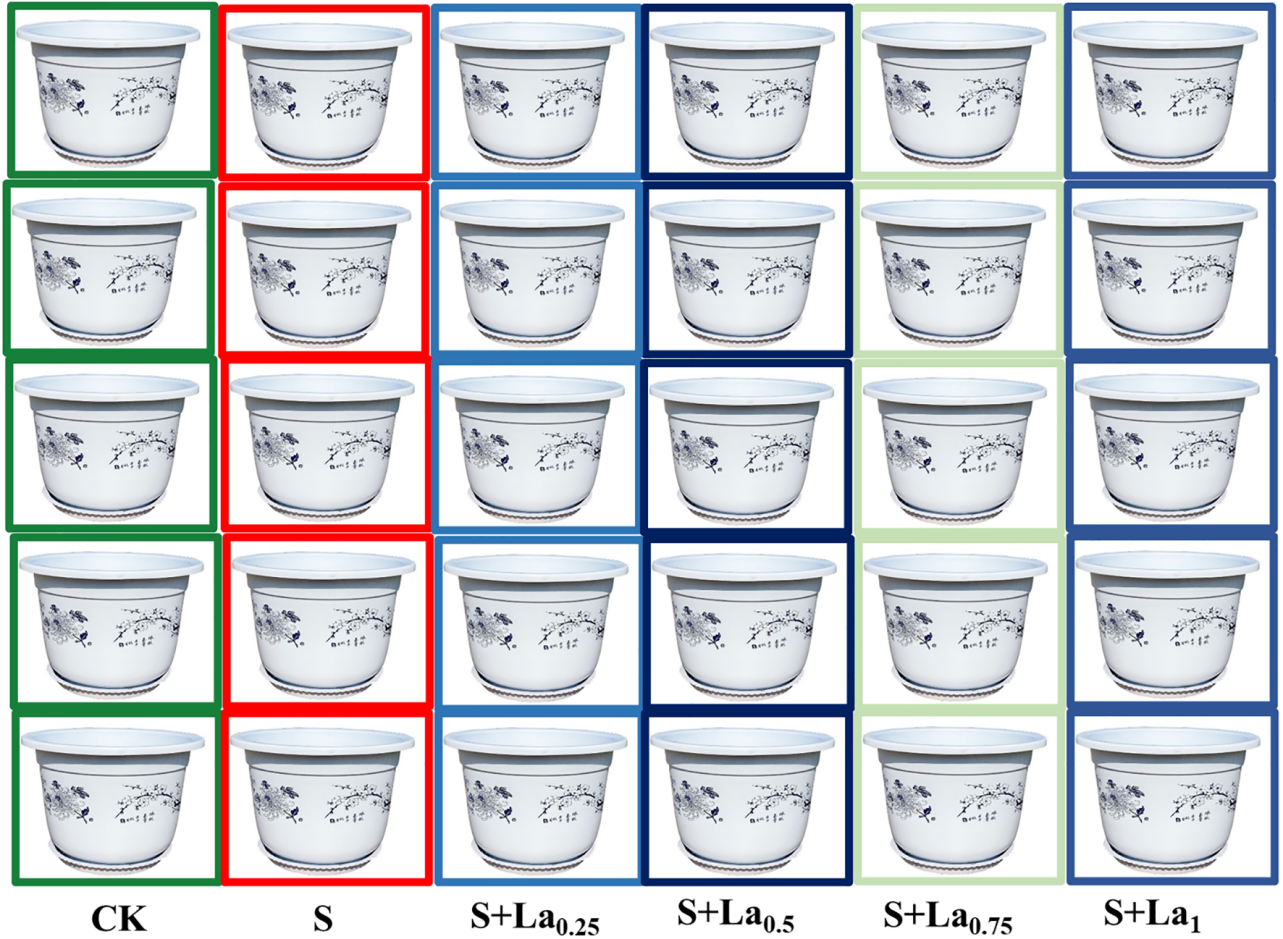
Detail of the treatments.

While the fifth leaf of the seedlings appeared, 25 pots were randomly selected for NaCl treatment, 300 mL of 160 mM NaCl solution was irrigated to each pot, and the other 5 pots (CK) were irrigated with the same amount of distilled water, once every two weeks, for a total of 5 times. After one week of the initial treatment, 5 concentrations of La(NO_3_)_3_ solution (0, 0.25, 0.5, 0.75 and 1.00 mM) were applied to the NaCl treatment groups, each concentration for 5 pots, 300 mL La(NO_3_)_3_ solution was added each time, once every two weeks, for a total of 5 times. So this study included 6 treatments (**Figure 2**). Salt treatment and La(NO_3_)_3_ treatment were applied alternately, with an interval of one week between the two kinds of treatments. The seedlings were harvested on September 17, 2021.

### 2.4 Determination of chlorophyll content and photosynthetic parameters

Determination of chlorophyll content: The relative chlorophyll content of 100 leaves of each treatment was measured with a chlorophyll content analyzer (SPAD-502, Chlorophyll Meter Model, Minolta Co., Ltd, Japan) on July 15, and their average value was calculated.

Determination of photosynthetic parameters: LI-6400 photosynthetic analyzer (LI-6400, Li-Cor Biosciences, Linconln, NE, USA) was used to determine the net photosynthetic rate (Pn), transpiration rate (Tr), stomatal conductance Gs) and intercellular CO2 concentration (Ci) of the third fully expanded leaf at the top of the seedlings of *G. uralensis* from 9:00 am to 11:00 am on July 15. The light intensity was set at 1200 μmol·m^2^·s^-1^. The CO_2_ concentration was set at 400 μmol·L^-1^, and the relative humidity and temperature were matched with the environmental conditions (22.2 ± 5.0% and 25.7 ± 4.0°C, respectively). 100 measurements per leaf, and the average values of each parameter were calculated.

### 2.5 Determination of antioxidant enzyme activity and malondialdehyde content

After the determination of photosynthetic parameters, 100 individuals of each treatment were randomly selected, and activities of superoxide dismutase (SOD), peroxidase (POD) and catalase (CAT) of their leaves and roots were determined by using the kits (SOD-BC0170, POD-BC0095, CAT-C017, Beijing Solarbio Science & Technology Co., Ltd., China). Malondialdehyde (MDA) content in leaves and roots was determined by thiobarbituric acid method (Xinping et al., 2024). The absorbance at 450, 532, and 600 nm were read using a spectrophotometer, the concentrations of MDA were calculated using the following equation:


$$\text{MDA }\left({\text{mol g}}^{-1}\text{FW}\right)\;=6.45\;\left({\text{A}}_{532}-{\text{A}}_{600}\right)－0.56{\text{A}}_{450}$$

### 2.6 Determination of root morphological indexes and biomass

On Sep 17th, 2021, all the plants were harvested, the fifth fully expanded leaf of each plant was collected from each treatment and scanned by Epson Digital Scanner (Expression 11000XL; Epson, Suwa, Japan) to calculate leaf area, and then were placed in a paper bag respectively. Stems and leaves in each treatment group were collected, and were placed in a marked paper bag. The roots were carefully rinsed with running water until there was no soil on their surface, and were scanned by the Epson digital scanner, root image analysis system software (Win RHIZO Pro2012b; regent Instruments Inc, Quebec City, QC, Canada) was used to measure total root length (TRL), maximum diameter of taproot (MTD), taproot length (TL), root volume (RV), root average diameter (AD), root branch number (Tips), etc. Then, the roots of each treatment were placed in paper bags. The bags containing leaves, stems and roots were heated in an oven at 105°C for 30 min, and then dried to constant weight at 70°C. The biomass of stems, leaves and roots of each plant were weighed, and their average value was calculated. The root-shoot ratio and specific leaf area were calculated according to the following formula:


Root-shoot ratio (R-S)=root dry weight/shoot dry weight

(Jia et al., 2024)


$$\text{Specific leaf area}:\text{SLA }\left({\text{cm}}^{2}\cdot {\text{g}}^{-1}\right)=\text{leaf area}/\text{leaf dry weight}$$

(Ihsan et al., 2024)

### 2.7 Determination of secondary metabolites of G. uralensis

A total of 0.500 g of dried and finely grounded root powder of each treatment was accurately weighed and transferred into a 10 mL centrifuge tube, then each tube was added with 5 mL methanol and extracted at room temperature for 24 h, and then the centrifuge tubes were placed in an ultrasonic extractor (KQ-300E, Kunshan Ultrasonic Instrument Co. Ltd., China) at room temperature for 2 h. The tubes were centrifuged at 12,000 rpm for 15 min, and the supernatant were collected. The concentrations of glycyrrhizic acid, glycyrrhetinic acid, liquiritin, liquiritigenin, and isoliquiritigenin in the supernatant were determined by high-performance liquid chromatography (HPLC) (Agilent 1200; Agilent Technologies, CA, USA). A UV-visible spectrophotometer (UV-1900, Shimadzu, Japan) was used to measure the total flavonoid concentration in the solution. The specific steps are as follows:

#### 2.7.1 Determination of glycyrrhizic acid, glycyrrhetinic acid, liquiritin, liquiritigenin and isoliquiritigenin

(1) Precisely 2.0 mg of each of the five secondary metabolite standards were weighed and transferred into a 10 mL volumetric flask respectively. The standards were dissolved in methanol and diluted to a final concentration of 200 μg·mL^-^¹. The stock solutions were further diluted with methanol to a series of standard solutions with concentrations of 1, 10, 50, 100, 250, 500, and 1000 ng·mL^-^¹. Ion peak area (Y) was plotted as the ordinate against concentration (X, ng·mL^-^¹) as the abscissa to construct standard curves. The linear equations for the five metabolites were as follows:

$$\text{glycyrrhizic acid}:\text{Y}=87.2\text{X}-3.76,{\text{ R}}^{2}=\;0.999,\;$$

$$\text{glycyrrhetinic acid}:\text{Y}=136.8\text{X}-122.4,{\text{ R}}^{2}=0.999,$$

$$\text{liquiritin}:\text{Y}=750.9\text{X}+356.9,{\text{ R}}^{2}=0.999,$$

$$\text{liquiritigenin}:\text{Y}=509.2\text{X}+925.6,{\text{ R}}^{2}=0.998,$$

$$\text{isoliquiritigenin}:\text{Y}=8775.1\text{X}+2617,{\text{ R}}^{2}=0.999$$

 The results demonstrated a good linear relationship between concentration and peak area in the range of 1–1000 ng·mL^-^¹ for all the five compounds.
(2) Concentration detection of glycyrrhizic acid, glycyrrhetinic acid, liquiritin, liquiritigenin and isoliquiritigenin: Specific methods refer to Jia et al (Jia et al., 2024). The content of the 5 substances per plant was calculated by combining the data with root biomass per plant.

#### 2.7.2 Determination of total flavonoid content

(1) 3.00 mg of liquiritin standard (Shanghai McLean Biochemical Technology Co., Ltd., China) was accurately weighed and fully dissolved in 5.0 mL of methanol, the resulting standard concentration was 0.4 mg·mL^-^¹. Subsequently, aliquots of 0, 25, 50, 100, 200, and 400 μL of liquiritin solution were taken into centrifuge tubes, to which 1 mL of methanol and 250 μL of 10% potassium hydroxide solution were added in turn. After standing for 5 min, the solutions were diluted to 5 mL with ethanol and thoroughly mixed. The absorbance was measured at 490 nm, and a standard curve was constructed. The regression equation was:

$$\text{Y}=0.026\text{X}-0.056,{\text{ R}}^{2}=0.999.$$

(2) Extraction of flavonoids: 0.500 g of *G. uralensis* root powder of each treatment was weighed and transferred into 10 mL centrifuge tubes, followed by the addition of 5 mL of methanol. The mixture was subjected to ultrasonic extraction for 1 h and then centrifuged at 12,000 rpm for 10 min. The supernatant was filtered using a 0.25 μm membrane. Then, 100 μL of the supernatant was mixed with 1 mL of 10% KOH solution and 1 mL of methanol, and the mixture was placed at room temperature for 5 min. The solution was then diluted to the mark in a 10 mL volumetric flask with methanol. Each treatment was repeated three times and mixed thoroughly before measurement.
(3) Determination of flavonoid concentration: A UV-visible spectrophotometer (UV-1900, Shimadzu, Japan) was used to measure the absorbance of the test solutions at 490 nm. The flavonoid concentration was calculated based on the regression equation. Each treatment was repeated 100 times, and the average value was calculated. The total flavonoid content per plant was determined by combining the results with root biomass per plant.

### 2.8 Data analysis:

#### 2.8.1 Statistical analysis and Parson correlation analysis

All experimental data were statistically analyzed using IBM SPSS 19.0 (IBM Corporation, Armonk, NY, USA) software. OriginPro 2024b was employed for plotting. All datasets were verified for normality using the D’Agostino and Pearson omnibus normality test. A one-way analysis of variance (ANOVA) with Turkey’s HSD *post-hoc* test (P< 0.05) was used to detect differences in photosynthetic gas exchange parameters, antioxidant enzyme activities, root growth, and the concentration of medicinal compounds across different treatments.

#### 2.8.2 Critic empowerment:

The specific calculation process is shown in Meira et al (Meira, 2015; XueYuan et al., 2023).

#### 2.8.3 NRBO-LSSVM-ABKDE coupling prediction model

**Figure 3:**
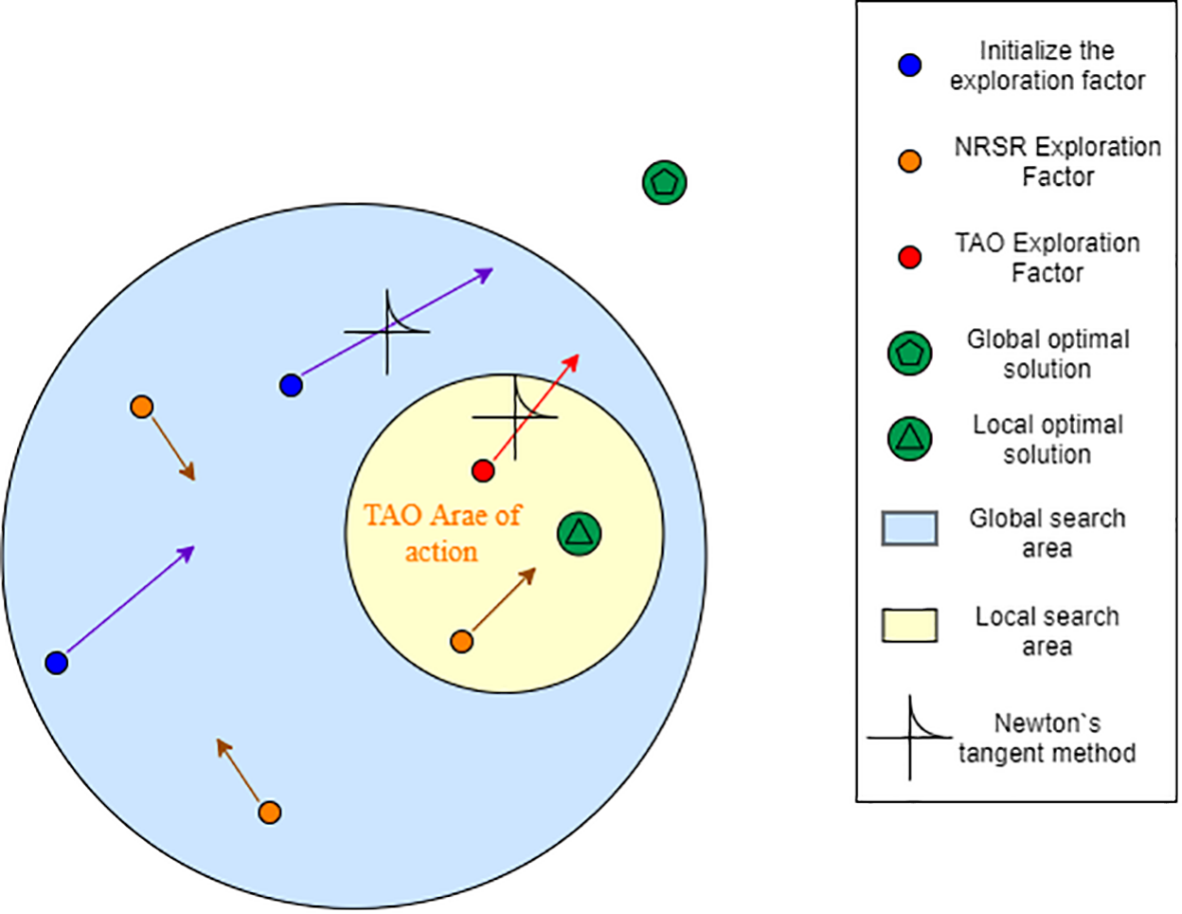
Principle diagram of NRBO algorithm.

(1) Newton-Raphson (NRBO) optimization algorithm:

**Figure 3** is the principle diagram of NRBO algorithm, it can be seen from the figure:

a. Population initialization
 Before the search, NRBO randomly generates a set of solution vectors in the solution space as the initial population. Each solution represents the solution of a potential problem. Each solution starts from different locations and explores the entire region from multiple directions.
 NRSR is based on the Newton iteration method, which is used to accelerate the speed of individuals in the population approaching the optimal solution. It guides the search direction by estimating the first and second derivatives (change trend) of the function, and combines the disturbance factor and the balance coefficient δ to improve the search ability. Generally speaking, when the population exploration factor searches for the optimal solution, it provides the correct direction of the optimal solution for the exploration factor and the fastest speed to approach the optimal solution, so that the exploration factor can find the optimal solution as soon as possible.
b. Newton-Raphson search rule (NRSR)
c. Trap avoidance operator (TAO)
 TAO is a mechanism to help individuals jump out of the local optimal trap. It generates a new solution vector through random perturbation and mixed solution information, and guides the population to jump from the current region to a potentially better region.

The specific formula derivation process is seen in the study of Ravichandran et al (Ravichandran et al., 2024).

(2) NRBO-LSSVM-ABKDE model calculation steps

**Figure 4:**
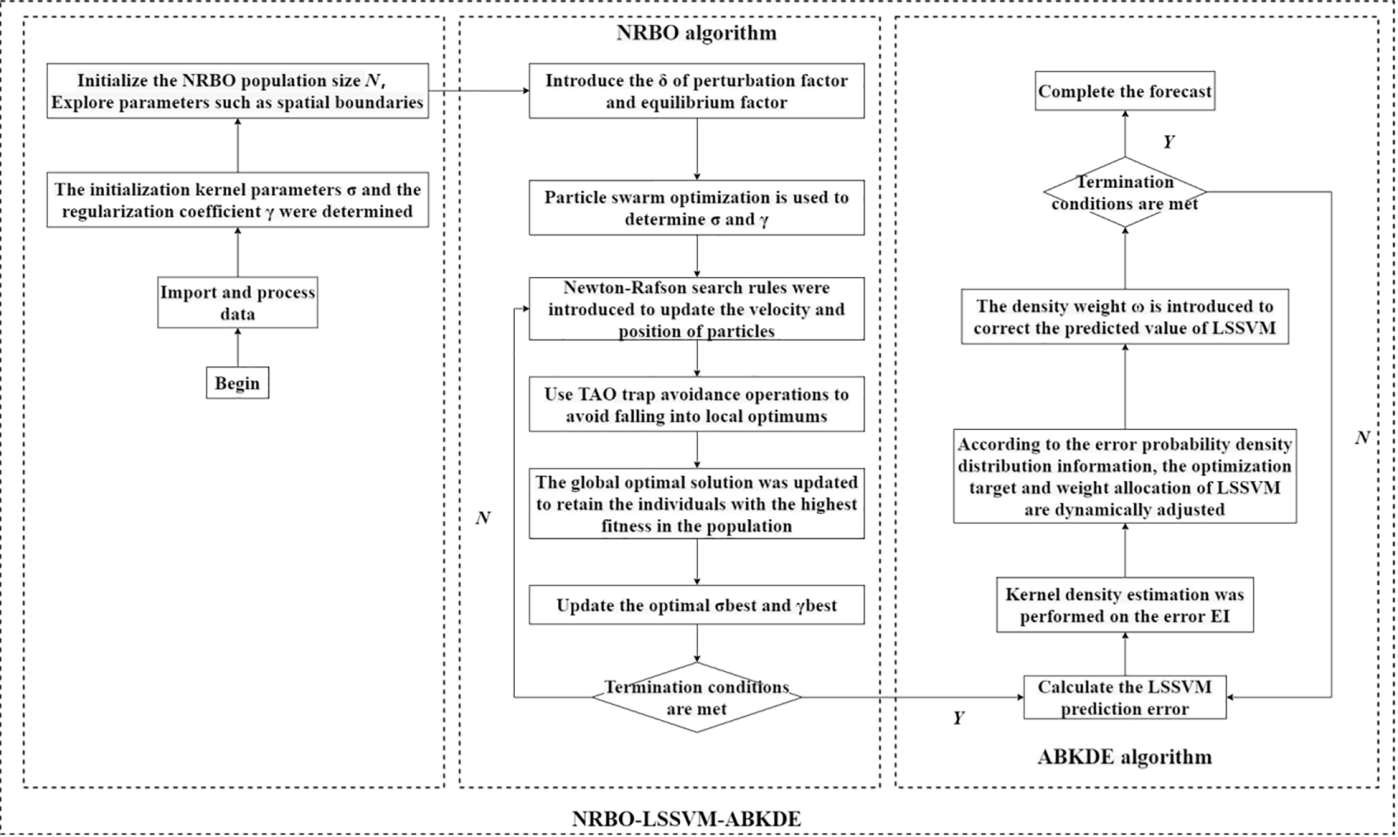
NRBO-LSSVM-ABKDE coupling prediction model.

**Figure 4** is the process of NRBO-LSSVM-ABRDE algorithm. From the figure, the process is as follows:

Firstly, import data and remove missing values to ensure the integrity and quality of data. The training set and the test set are divided, and the data of the training set and the test set are normalized to ensure that all features are within the same scale range.

Set the relevant parameters of the NRBO (Newton-Raphson) algorithm, including the population size N, the maximum number of iterations (Maxlt), the upper and lower bounds of the exploration space (LU and LB), and the optimization dimension, the optimized dimensions are γ and σ, which determine the model structure of LSSVM.

In the NRBO algorithm, the position of the particle represents the hyper-parameters γ and σ of the LSSVM, and these two hyper-parameters are determined by particle swarm optimization. The particle fitness formula is as follows:


$$f\left(P_{i}\right)=MSE_{test}\left(\gamma_{i},\sigma_{i}\right)$$

Pi represents the position of the particle, including the γ value and σ value of the current particle.

In each iteration, the particle calculates the step size according to its current position and velocity, and updates the position through the Newton-Raphson search rule. In order to avoid falling into local optimum, trap avoidance operation is used. The velocity update formula of the particle is as follows:


$$v_{i}^{new}=\omega \cdot v_{i}+c_{1}\cdot \gamma_{1}\cdot \left(p_{i}^{best}-p_{i}\right)+c_{2}\cdot \gamma_{2}\cdot \left(g_{i}^{best}-p_{i}\right)$$

Among them, ω is the inertia weight, which controls the inertia of the particle to the upper position, C1 and C2 are learning factors, which determine the pulling force of particles to the local optimal solution and the global optimal solution. γ1 and γ2 are random numbers, the position update formula of the particles is as follows:


$$P_{i}^{new}=p_{i}+v_{i}^{new}$$

If the fitness of a particle is better than the current global optimal solution, that is, the global optimal solution pbest and gbest (that is, σ and γ) are updated, and the optimal σ and γ are finally determined by multiple iterations. The update formula is as follows:


$$g^{best}=\arg\underset{p_{i}}{min}f\left(p_{i}\right)$$

$$p^{best}=\arg\underset{p_{i}}{min}f\left(p_{i}\right)$$

The training set and the optimal hyperparameters (i.e., σbest and γbest) are used to initialize the LSSVM model and train it. The training process adjusts the model parameters by minimizing the objective function. The objective function is as follows:


$$\underset{\omega ,b,\in }{min}\frac{1}{2}w^{T}w+\frac{\gamma }{2}\sum_{i=1}^{n}\in_{i}^{2}$$

According to the probability density distribution information obtained by ABKDE, the optimization objective and weight distribution of LSSVM are dynamically adjusted, and the fitting effect of the model is optimized by introducing the density weight ω (determined by the density estimation value of ABKDE).

The final LSSVM model is trained by the optimal parameter configuration and outputs the prediction results.

## 3 Result

### 3.1 Effects of La(NO_3_)_3_ on physiological characteristics of G. uralensis under salt stress

#### 3.1.1 Effects of La(NO_3_)_3_ on photosynthetic parameters

Under the condition without adding La(NO_3_)_3_, NaCl treatment significantly reduced the photosynthetic parameters of *G. uralensis*. Compared with the CK, the specific leaf area, net photosynthetic rate, stomatal conductance, intercellular CO_2_ concentration, and transpiration rate of the S treatment were significantly reduced by 21%, 9%, 42%, 27%, 24% and 59%, respectively. It indicated that salt stress caused systemic damage to the photosynthetic physiology of licorice.

The application of La(NO_3_)_3_ promoted photosynthesis potential of the licorice. With increasing concentrations of lanthanum nitrate from 0.25 to 0.75 mM, the above parameters significantly improved, reaching optimal values at La(NO_3_)_3_ concentration of 0.75 mM. Compared with the S treatment, the above mentioned indicators in the treatment of S+La0.75 increased by 70%, 47%, 61%, 36%, 42% and 57%, respectively (**Table 1**). These results indicated that 0.75 mM lanthanum nitrate effectively promoted stomatal opening (a substantial increase in Gs, Tr, Ci) and leaf growth expansion (a substantial increase in SLA) under salt stress, and may comprehensively and significantly reverse the photosynthetic inhibition effect of salt stress by protecting the photosynthetic apparatus, enhancing stress resistance and other non-stomatal mechanisms, so that the net photosynthetic rate was restored by nearly half.

**Table 1:** Effects of La(NO_3_) _3_ on photosynthetic parameters of *G. uralensis* under NaCl stress.

Treatments	SLA (cm^2^·g^-1^)	Chl (SPAD)	Pn (μmol·m^-^²·s^-^¹)	Gs (mol·m^-^²·s^-^¹)	Ci (µmol·mol^-^¹)	Tr (mmol·m^-^²·s^-^¹)
CK	13.8 ± 0.5^d^	26.6 ± 1.4^d^	10.9 ± 0.7^b^	71.9 ± 5.0^b^	194.1 ± 16.6^b^	3.3 ± 0.2^a^
**S**	**11.0± 0.6^e^**	**24.2 ± 0.1^f^**	**6.3 ± 0.2^f^**	**52.2 ± 1.7^f^**	**148.3 ± 6.3^e^**	**1.4 ± 0.1^e^**
S + La_0.25_	16.5± 0.1^c^	28.4 ± 0.3^c^	8.0 ± 0.9^e^	63.3 ± 2.4^d^	191.3 ± 13.7^b^	1.5 ± 0.1^d^
S + La_0.5_	17.2± 0.3^b^	30.8 ± 0.7^b^	9.2 ± 1.0^c^	67.9 ± 3.7^c^	186.5 ± 1.2^c^	1.6 ± 0.1^c^
**S + La_0.75_**	**18.6± 0.1^a^**	**35.7 ± 0.6^a^**	**10.1± 0.9^a^**	**70.5 ± 3.4^a^**	**208.9 ± 18.5^a^**	**2.1 ± 0.1^b^**
S + La_1.0_	13.7± 0.3^d^	25.6 ± 0.3^e^	8.4 ± 1.0^d^	56.4 ± 4.2^e^	178.3 ± 11.5^d^	1.5 ± 0.1^c^

Different letters indicated significant difference between the treatments (mean ± standard deviation, *P*< 0.05).

The crude part of the table was NaCl stress and the effect of applying 0.75 mM lanthanum nitrate on the photosynthetic characteristics of Glycyrrhiza uralensis under NaCl stress. By comparison, the mitigation effect of lanthanum nitrate on photosynthesis of Glycyrrhiza uralensis was clarified.

#### 3.1.2 Effects of La(NO_3_)_3_ on antioxidant enzyme activity and MDA content

**Figure 5:**
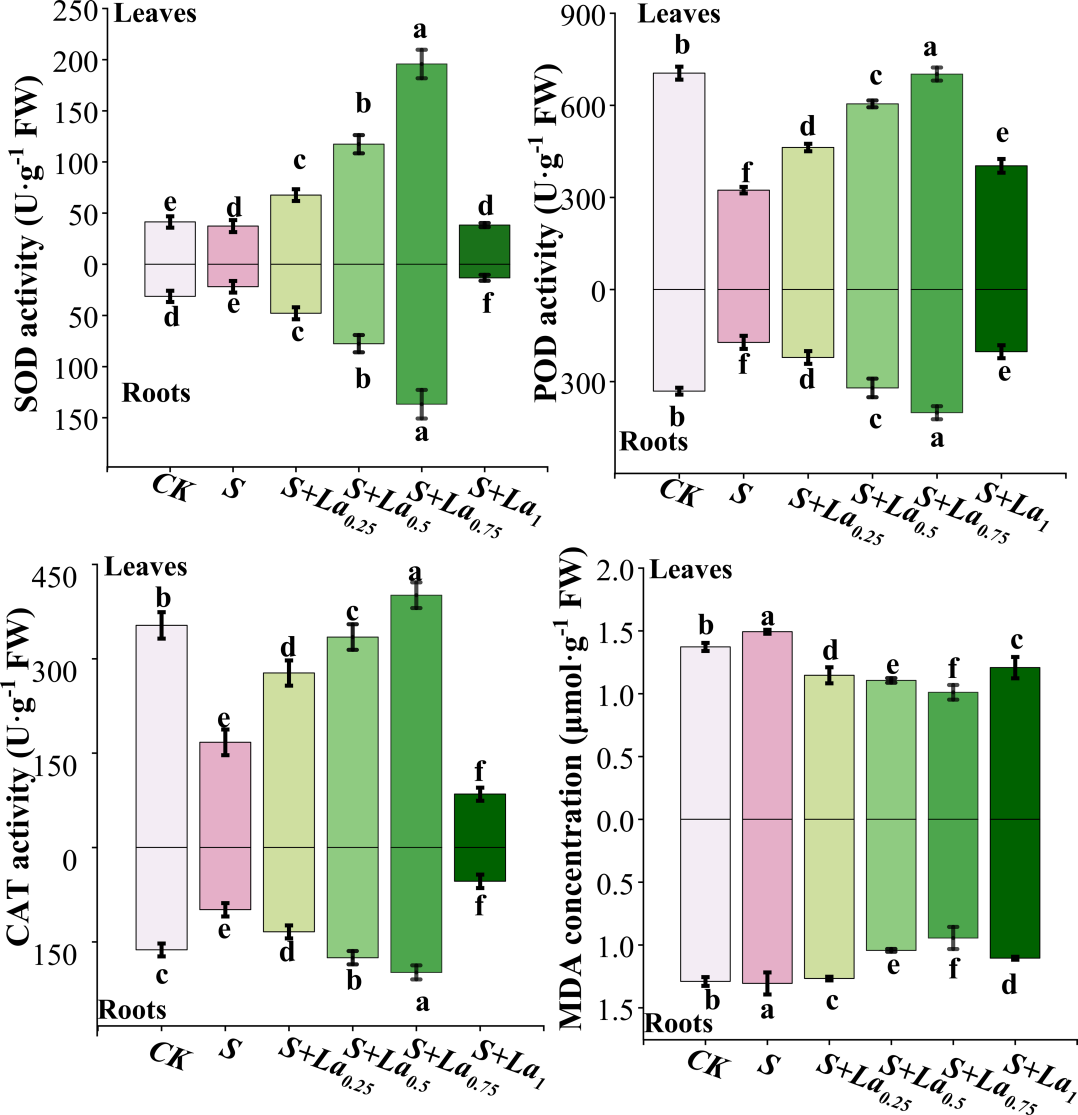
The effects of La ( NO3 ) 3 on the activities of SOD, POD, CAT and the content of MDA in Glycyrrhiza uralensis under salt stress. Different letters indicated significant difference between the treatments (mean ± standard deviation, *P*< 0.05).

Compared with CK, the activities of SOD, POD and CAT in leaves of *G. uralensis* under S treatment were significantly decreased by 11%, 54% and 53% in turn, and the content of MDA was increased by 9%. The variation in the antioxidant enzyme activity and MDA content in roots were similar to those in leaves. The activities of the 3 enzymes in the S treatment were significantly lower than the CK by 30%, 49% and 39%, respectively (**Figure 5**). This indicates that NaCl treatment breaks the balance of active oxygen metabolism in *G.uralensis* by inhibiting the activity of key antioxidant enzymes, resulting in a large accumulation of ROS, which in turn attacks the cell membrane and causes membrane lipid peroxidation.

Application of La(NO_3_)_3_ significantly promoted the activities of these enzymes both in leaves and roots, while reducing the level of MDA. With the increase of La(NO_3_)_3_ concentration from 0.25 to 0.75 mM, the enzyme activity increased significantly, and the maximum value appeared in the treatment of S + La_0.75_. Compared with the S treatment, activity of the 3 enzymes in leaves under the S+La_0.75_ treatment increased by 424%, 117% and 140%, respectively, and its MDA content decreased by 33%. The enzyme activity in roots increased by 525%, 133% and 102%, respectively, and the MDA content reduced by 29% (**Figure 5**). This indicated that the application of La(NO_3_)_3_ could effectively activate the antioxidant defense system of *G.uralensis* and reduce the degree of membrane lipid peroxidation, thus significantly alleviating the oxidative damage caused by NaCl stress, and its mitigation effect was concentration-dependent.

#### 3.1.3 Effect of La(NO_3_)_3_ on the relative content of medicinal secondary metabolites

Compared with the CK group, content of these substances in the S group decreased by 17%, 3%, 25%, 31%, 49% and 21% (**Table 2**). This indicated that Nacl treatment severely inhibited the synthesis and accumulation of secondary metabolites in the roots of *G.uralensis*, and weakened its chemical defense ability and environmental adaptability. However, the addition of La(NO_3_)_3_ significantly increased the content of these components in the roots. Compared with the CK, contents of these components in the S + La0.75 group increased by 64%, 129%, 83%, 99%, 37% and 58%, respectively. And, they increased by 99%, 133%, 141%, 181%, 167% and 98%, respectively compared with the S group (**Table 2**). This proved that the application of La(NO_3_)_3_ not only reversed the inhibitory effect of salt stress on secondary metabolism, but also activated it to the ‘ overcompensation ‘ state far beyond the normal level, which greatly enhanced the chemical defense and adaptability of *G.uralensis*.

**Table 2:** Effects of La (NO_3_)_3_ on the content of main medicinal components of *G. uralensis* under NaCl stress.

Treatment (mM)	Glycyrrhizic acid (mg·plant^-1^)	Glycyrrhetinic acid (ng·plant^-1^)	Liquiritin (mg·plant^-1^)	Liquiritigenin (ng·plant^-1^)	Isoliquiritigenin (ng·plant^-1^)	Total flavonoids (mg·plant^-1^)
CK	3.1 ± 0.8^c^	87.3 ± 4.1^c^	1.6 ± 0.4^d^	325.7 ± 4.7 ^c^	114.9 ± 13.7^c^	1.5 ± 0.2^c^
**S**	**2.6 ± 0.5^e^**	**85.6 ± 4.0^e^**	**1.2 ± 0.2^d^**	**228.9 ± 12.5^d^**	**58.6 ± 9.3^e^**	**1.2 ± 0.1^e^**
S + La_0.25_	2.9 ± 0.5^d^	101.3 ± 6.0^d^	1.5 ± 0.3^c^	321.4 ± 11.4^c^	79.3 ± 7.7^d^	1.4 ± 0.1^d^
S + La_0.5_	4.0 ± 0.2^b^	161.1 ± 6.4^b^	2.4 ± 0.3^b^	518.8 ± 37.6^b^	127.3 ± 5.8^b^	2.0 ± 0.2^b^
**S + La_0.75_**	**5.1 ± 0.4^a^**	**199.4 ± 3.1^a^**	**3.0 ± 0.6^a^**	**644.5 ± 51.9^a^**	**156.1 ± 18.3^a^**	**2.4 ± 0.4^a^**
S + La_1.0_	2.2 ± 0.5^f^	69.8 ± 3.2^f^	0.8 ± 0.1^e^	202.0 ± 7.6^e^	40.6 ± 3.0^f^	1.1 ± 0.1^f^

Different letters indicated significant difference between the treatments (mean ± standard deviation, *P*< 0.05).

The crude part of the table is NaCl stress and the effect of applying 0.75 mM lanthanum nitrate on the content of the main pharmacological components of Glycyrrhiza uralensis under NaCl stress. By comparison, it can be clarified that lanthanum nitrate can significantly increase the content of pharmacologically active components of Glycyrrhiza uralensis under salt stress.

### 3.2 Effect of La(NO_3_)_3_ on root growth of G. uralensis under salt stress

#### 3.2.1 Effects of La(NO_3_)_3_ on root morphology

**Figure 6:**
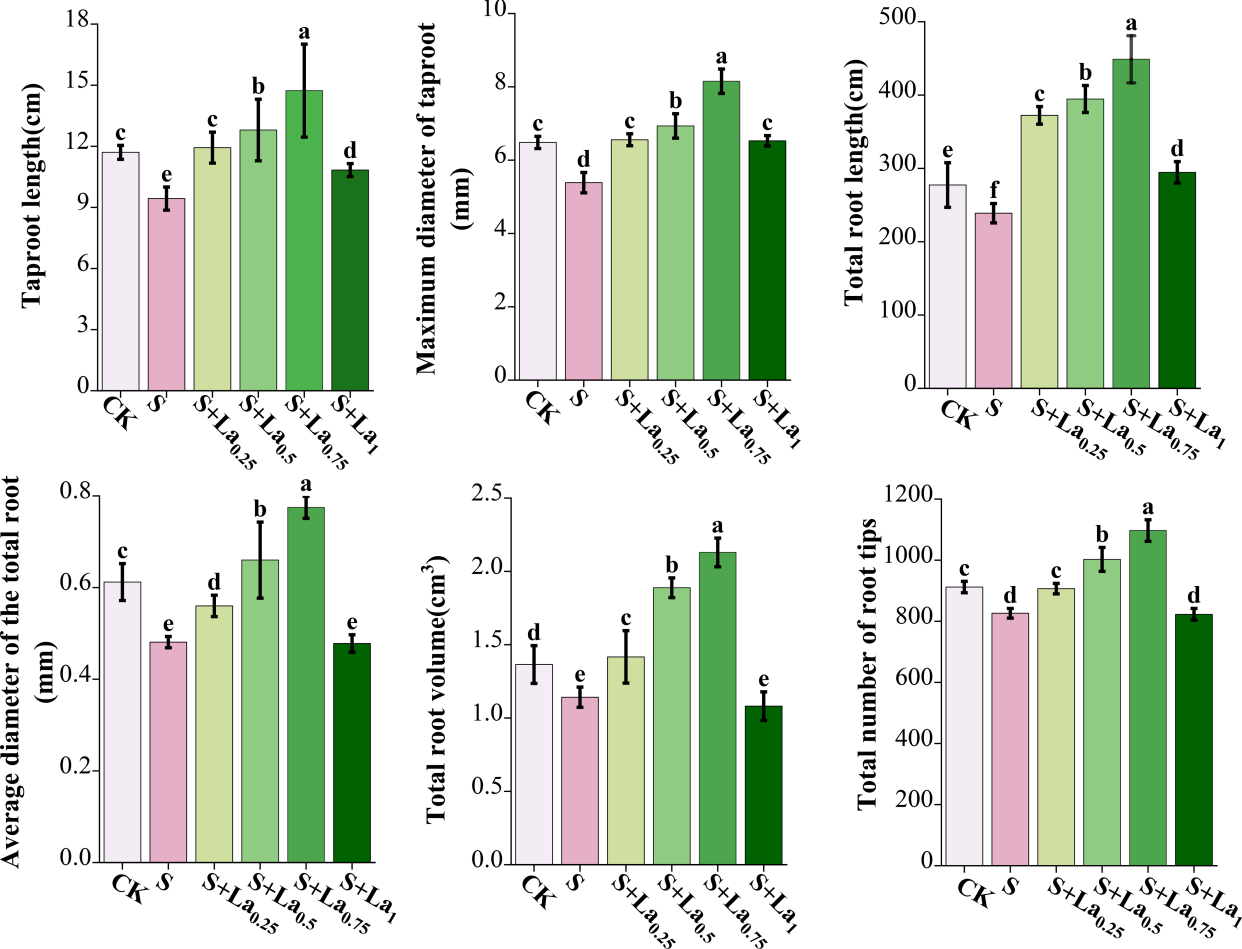
The effects of La ( NO3 ) 3 on the taproot length, maximum diameter of taproot length, total root length, average root diameter, total root volume and total root tip number of G.uralensis roots under NaCl stress. Different letters indicated significant difference between the treatments (mean ± standard deviation, *P*< 0.05).

**Figure 7:**
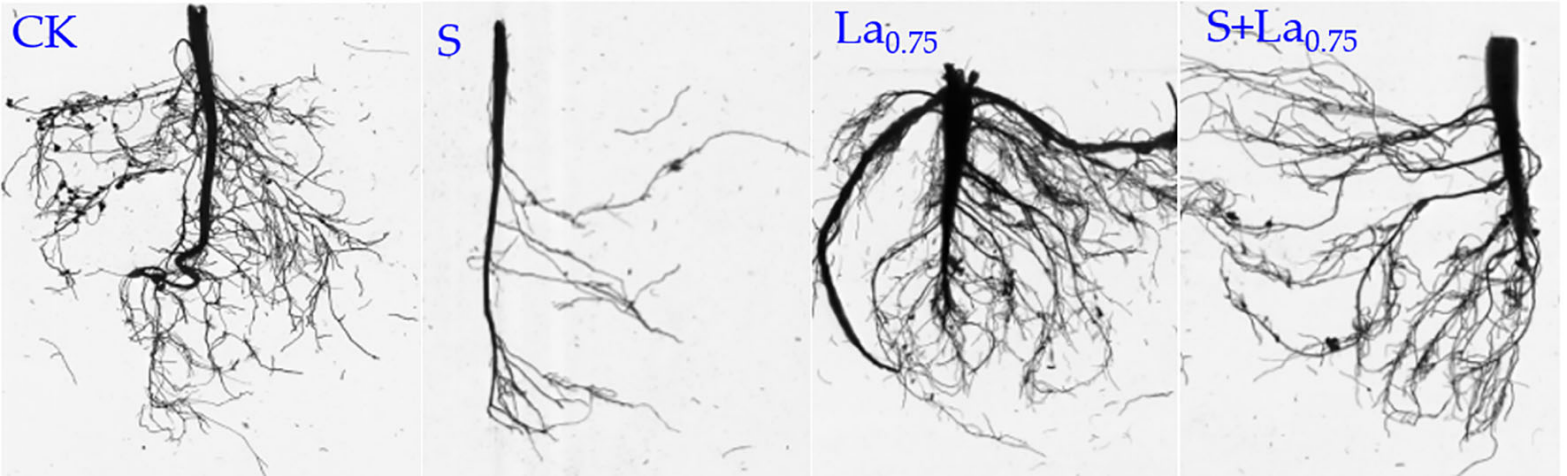
Effects of La (NO_3_) _3_ on root morphology of *G. uralensis* seedlings under NaCl stress.

Compared with CK, the taproot length, maximum main root diameter, total root length, average root diameter, total root volume and total root tip number of the S treatment were significantly reduced by 19%, 18%, 15%, 21%, 17% and 9% (**Figure 6**, **7**).

The application of La(NO_3_)_3_ significantly improved the above indicators. With the increase of lanthanum nitrate concentration, these indicators increased significantly under the treatments of 0.25-0.75 mM La(NO_3_)_3_, and reached the maximum under the treatment of 0.75 mM La(NO_3_)_3_. Compared with the S treatment, the above indicators of the S+La_0.75_ group increased by 56%, 52%, 89%, 62%, 86% and 33% (**Figure 6**, **7**).

The above results showed that salt treatment caused systematic development inhibition of *G.uralensis* roots, which seriously damaged its absorption function and anchoring ability, which was one of the fundamental reasons for the limited growth of aboveground parts of plants. However, the application of La(NO_3_)_3_ effectively reversed the inhibitory effect of salt treatment on root development, not only restored root growth, but also exceeded the normal level in some aspects, which greatly enhanced the rhizosphere competitiveness of plants.

#### 3.2.2 Effects of La(NO_3_)_3_ on the biomass

**Figure 8:**
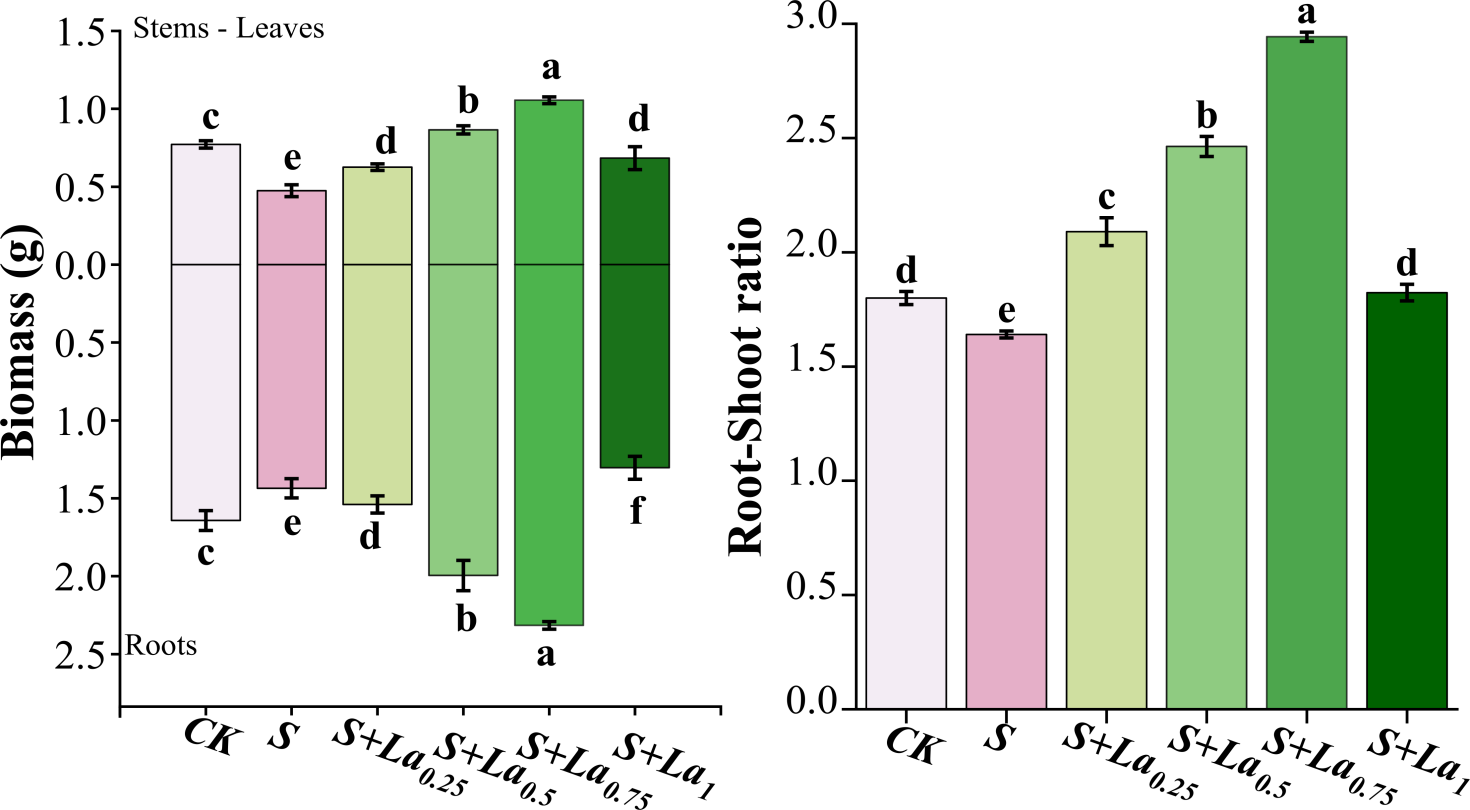
Effects of La ( NO3 ) 3 on root biomass, stems-leaves biomass and root-shoot ratio of Glycyrrhiza uralensis under NaCl stress. Different letters indicated significant difference between the treatments (mean ± standard deviation, *P*< 0.05).

Compared with the CK, the above parameters of S group decreased by 13%, 38% and 10%, respectively, indicating that salt stress (S) substantially inhibited the growth of *G. uralensis*, particularly root development. The application of La(NO_3_)_3_ effectively mitigated this salt stress, as it obviously promoted stem-leaf biomass, root biomass and root-shoot ratio of *G. uralensis*. With the increasing of lanthanum concentration, these indicators increased significantly under 0.25-0.75 mM treatment conditions, reaching peak values at 0.75 mM.This clear dose-dependent response suggests that lanthanum, within this concentration range, acts as a potent stimulator of growth and root allocation under salt stress. Compared with the CK, the above indicators of the S+La_0.75_ treatment increased by 41%, 37% and 64%, respectively. Remarkably, these indicators of the S+La_0.75_ group increased by 61%, 122% and 79% compared with the S group (**Figure 8**). This demonstrates that the 0.75 mM La(NO_3_)_3_ treatment not only fully reversed the detrimental effects of salt stress but also significantly enhanced growth and root-shoot partitioning beyond the levels observed in the unstressed control (CK). The exceptionally high increase in root biomass (122%) compared to the stressed group highlights lanthanum’s pronounced effect on promoting root system development under salinity, which is crucial for water and nutrient uptake in adverse conditions.

### 3.3 Correlation

**Figure 9:**
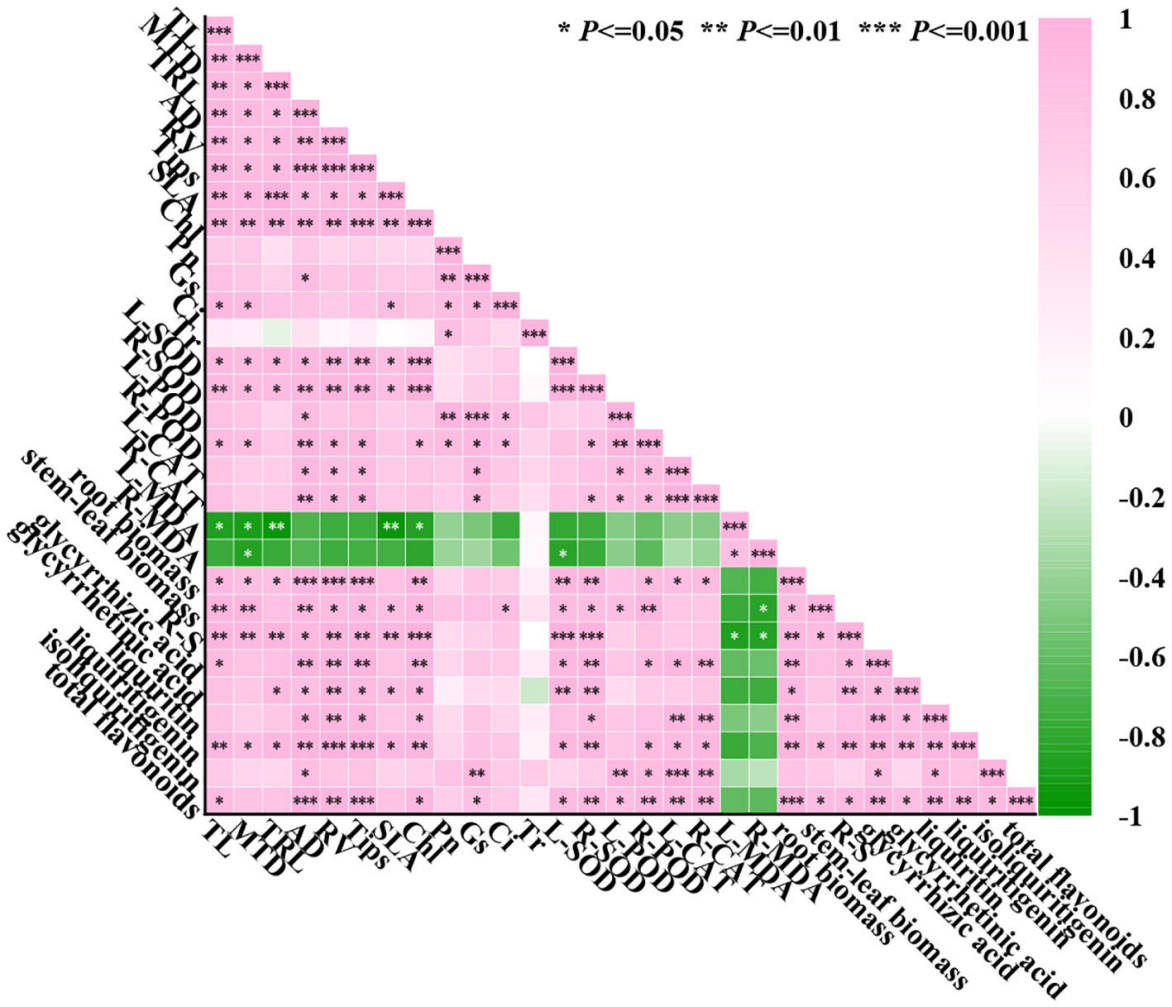
Pearson correlation heat map of the effect of La(NO_3_)_3_ on 29 indexes of *G. uralensis* under NaCl stress. Pink: means positive correlation, that is, there is a positive correlation between the two indicators. The closer the value is to 1, the stronger the correlation is. Green: indicates a negative correlation, that is, there is a negative correlation between the two indicators. The closer the value is to -1, the stronger the negative correlation is.

Using SPSS analysis software to analyze 29 indicators, as shown in **Figure 9**, there is a strong correlation between the indicators. Most of the growth indexes were positively correlated with antioxidant enzyme activities (such as L-SOD, L-CAT, R-POD, etc.), indicating that the antioxidant capacity of *G. uralensis* was enhanced by the regulation of lanthanum nitrate, which was helpful to alleviate salt stress to growth of the licorice. The growth indexes were negatively correlated with the level of malondialdehyde (MDA), indicating that La(NO_3_)_3_ may sustain the normal growth of cells by reducing membrane lipid peroxidation damage. There was a significant positive correlation between antioxidant enzymes, indicating that they may synergistically improve the antioxidant capacity of *G. uralensis*. The activity of antioxidant enzymes was significantly negatively correlated with MDA, indicating that the higher the activity of antioxidant enzymes, the lower the degree of membrane lipid peroxidation. Photosynthetic indexes were significantly positively correlated with other indexes except MDA, indicating that La(NO_3_)_3_ effectively improved the function of photosynthetic system of the licorice under NaCl stress and significantly promoted growth and accumulation of secondary metabolites.

### 3.4 Construction of comprehensive influence index system of G. uralensis plant

Based on the study of the effects of La(NO_3_)_3_ in the above experiments on photosynthesis, anti-oxidative stress, growth parameters and relative content of secondary metabolites of *G.uralensis* under salt stress, in order to further determine the effects of photosynthesis and anti-oxidative stress mechanism on the growth and pharmacological active substances of *G.uralensis*, the above 29 indicators such as SOD, POD, CAT and MDA were standardized, and then weighted by Critic method. The results are shown in **Table 3**. Through the weight distribution of Critic method, the 29 indexes after weighting are transformed into four comprehensive index systems of photosynthesis system, antioxidant stress system, growth system and secondary metabolite system, and the data set of *Glycyrrhiza uralensis* plants is constructed by combining the weight distribution. The results are shown in **Table 4**, which provides a theoretical and data basis for subsequent prediction model experiments.

**Table 3:** Critic weights of each index.

Index	CK	S	S+La_0.25_	S+La_0.5_	S+La_0.75_	S+La_1_
	Critic weight (%)					
root biomass	0.1	0.1	0.1	0.1	0.1	0.1
stem-leaf biomass	0.1	0.1	0.1	0.1	0.1	0.1
R-S	0.1	0.2	0.2	0.2	0.2	0.2
TL	1.0	0.9	1.0	0.9	0.9	0.9
MTD	0.6	0.5	0.5	0.5	0.5	0.6
TRL	24.1	23.0	28.1	29.4	30.0	25.8
AD	0.0	0.0	0.1	0.0	0.0	0.0
RV	0.1	0.1	0.0	0.1	0.1	0.1
Tips	73.8	75.2	70.0	68.7	68.1	72.3
SLA	4.1	5.0	4.9	4.9	5.6	5.1
Chl	8.3	9.5	9.5	9.9	8.9	7.7
Pn	3.4	2.9	3.4	3.1	2.9	2.8
Gs	24.7	22.7	22.4	23.1	22.5	16.9
Ci	58.5	59.5	59.4	58.6	59.5	66.9
Tr	1.0	0.5	0.5	0.5	0.6	0.6
L-SOD	2.5	4.3	5.8	8.0	10.8	5.5
R-SOD	2.1	2.5	4.6	5.0	6.8	1.6
L-POD	44.5	37.1	34.6	36.5	31.3	47.9
R-POD	17.3	22.3	17.3	19.4	20.3	26.9
L-CAT	22.9	21.8	24.7	21.4	21.3	10.9
R-CAT	10.5	11.6	12.9	9.6	9.3	6.9
L-MDA	0.1	0.2	0.0	0.1	0.1	0.2
R-MDA	0.1	0.1	0.0	0.1	0.1	0.1
glycyrrhizic acid	57.7	57.9	57.7	50.7	53.7	69.2
glycyrrhetinic acid	1.8	2.2	2.0	2.1	2.7	2.0
liquiritin	31.2	32.3	32.6	37.9	34.1	21.8
liquiritigenin	6.9	6.1	6.0	7.4	7.6	5.7
isoliquiritigenin	2.4	1.5	1.7	1.8	2.0	1.3
total flavonoids	0.0	0.0	0.0	0.0	0.0	0.0

**Table 4:** Comprehensive weights of different systems.

System	CK	S	S+La_0.25_	S+La_0.5_	S+La_0.75_	S+La_1_
	Comprehensive weight					
Anti-oxidative stress	470.7	208.7	428.9	384.6	435.9	262.8
	441.8	204.9	466.7	402.2	432.6	249.6
	472.6	215.4	423.2	383.8	425.6	236.8
	480.6	209.0	406.8	361.1	420.7	262.9
	456.0	203.2	433.2	390.5	436.1	263.2
Photosynthesis	134.5	103.0	131.5	129.1	144.8	131.8
	146.1	103.5	122.2	131.4	135.0	105.9
	138.6	100.7	132.9	139.6	144.3	130.8
	131.1	102.5	141.5	131.9	138.9	126.7
	125.9	96.3	136.2	134.1	150.3	119.0
Growth	740.4	675.9	739.0	805.2	881.5	670.7
	729.2	659.4	707.3	851.8	880.5	741.2
	690.8	685.4	718.0	791.7	960.2	672.3
	685.1	640.7	745.3	767.6	908.7	655.4
	750.6	697.6	728.3	797.5	870.2	807.0
Pharmacological activity	1421.1	1327.5	1424.1	1491.0	1645.4	1284.1
	1442.1	1208.4	1493.2	1549.4	1714.8	1219.6
	1389.1	1358.9	1451.9	1467.0	1522.2	1231.9
	1295.8	1256.5	1422.9	1585.1	1684.3	1385.2
	1471.9	1335.6	1460.3	1450.3	1606.6	1319.2

### 3.5 Dynamic prediction of comprehensive index of G. uralensis based on coupling model

**Figure 10:**
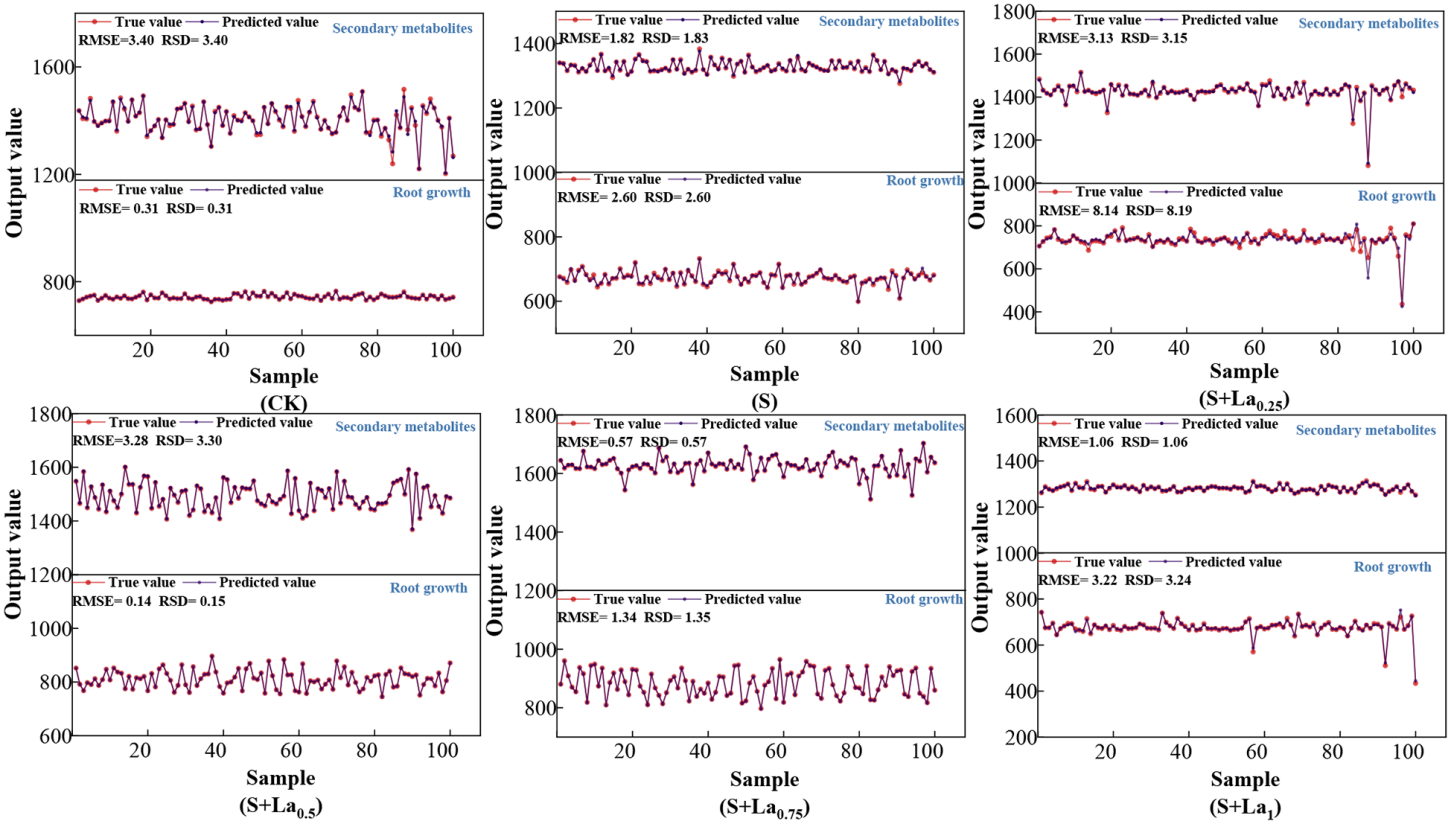
The prediction results of the NRBO-LSSVM-ABKDE prediction model under different treatments.

Based on the data set constructed in Section 2.4, the NRBO-LSSVM-ABKDE coupling prediction model was introduced in this study. The photosynthesis system and the anti-oxidative stress system were used as input variables, and the growth system and the secondary metabolite system were used as output variables, respectively. The relevant indicators of *G. uralensis* under different treatment conditions were predicted and analyzed. The results are shown in **Figure 10**.

It can be seen from the prediction results of each treatment group in the figure that the predicted value of the model is basically consistent with the overall trend of the measured value, and the residual value (RSD) fluctuates less, indicating that the model has high accuracy and stability. Specifically, under the CK group, the model was robust to the prediction of growth indicators and medicinal active ingredients, the predicted value was highly fitted with the measured value, and the residual fluctuation was small, which reflected that the physiological metabolic process of licorice had strong regularity under stress-free conditions, and the model capture ability was strong. In group S, some indexes fluctuated greatly, especially in the aspect of pharmacological active components, but the model could still restore its changing trend and showed strong adaptability. Under the treatment of different concentrations of exogenous lanthanum nitrate, the model prediction effect was generally good, especially in the S + La0.75 group, the fitting effect was particularly significant, which verified the stability of the model under the input of multiple sets of variables, and it could not only accurately reflect the growth dynamics of *Glycyrrhiza uralensis* and the accumulation trend of medicinal components, but also effectively apply to the growth adaptability modeling of *Glycyrrhiza uralensis* under complex environmental stress and exogenous regulation conditions.

### 3.6 Comparison of multi-model prediction results

#### 3.6.1 Model fitting analysis

**Figure 11:**
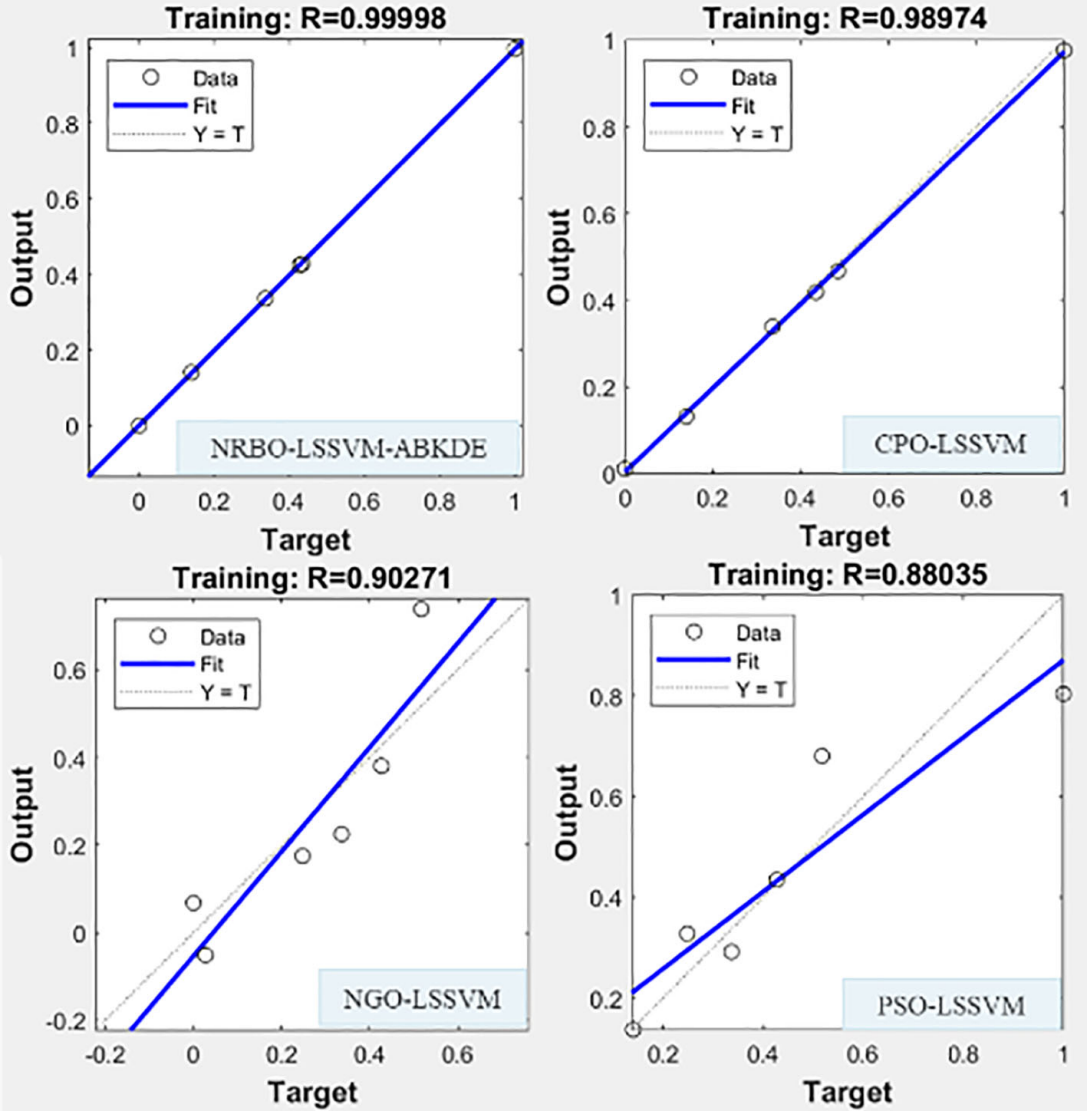
Four prediction models (NRBO-LSSVM-ABKDE, CPO-LSSVM, NGO-LSSVM, PSO-LSSVM) were used to fit the growth system of *G.uralensis* in S + La_0.75_ group.

**Figure 12:**
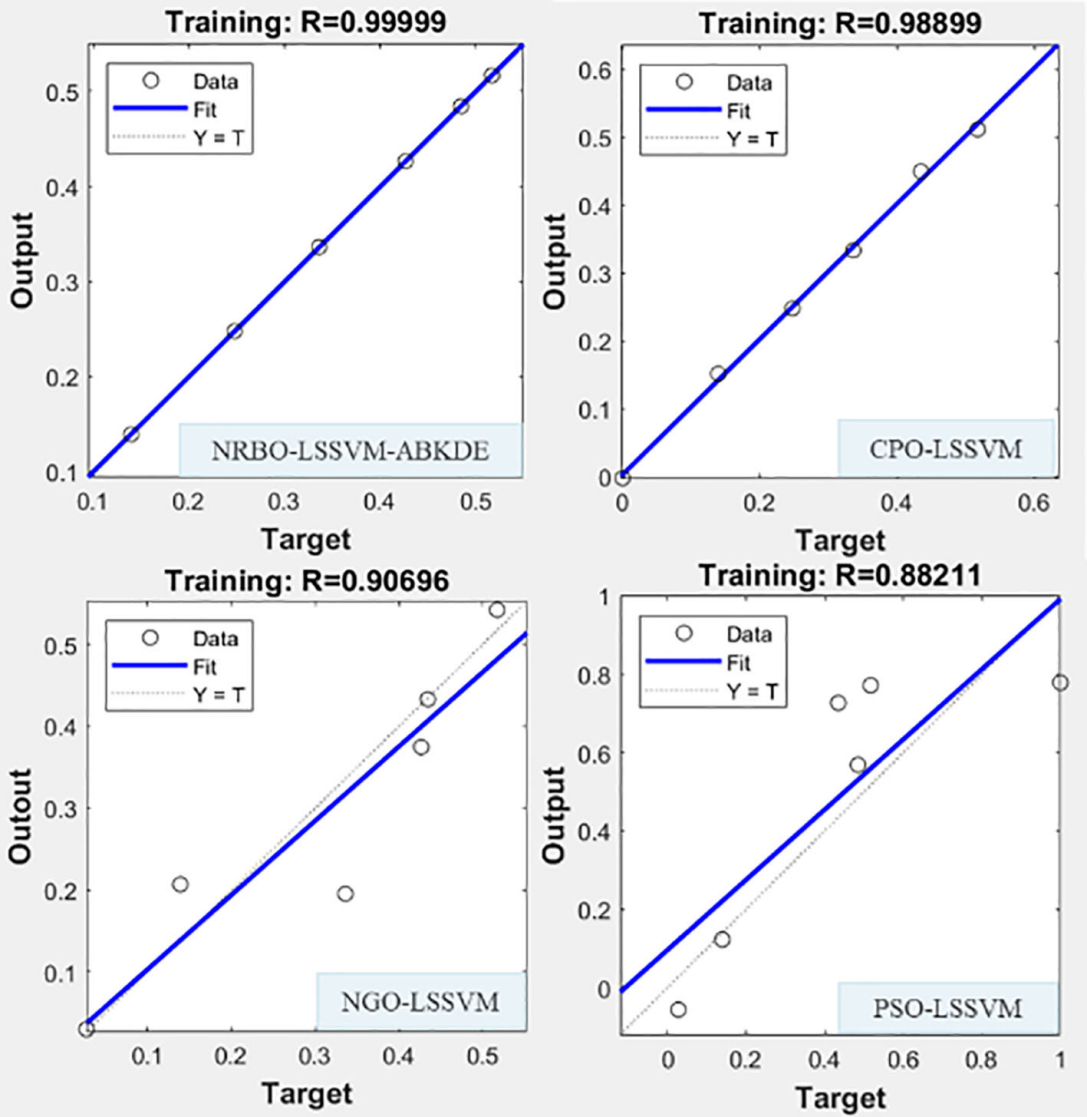
Four prediction models (NRBO-LSSVM-ABKDE, CPO-LSSVM, NGO-LSSVM, PSO-LSSVM) were used to fit the growth system of *G.uralensis* in in S + La_0.75_ group.

In order to further verify the comprehensive performance of NRBO-LSSVM-ABKDE model in the prediction of different indexes of *G.uralensis*, this study selected some data sets of S + La_0.75_ group, and compared them with three mainstream optimization algorithm models (CPO-LSSVM, PSO-LSSVM, NGO-LSSVM), and compared their fitting effects in the prediction of growth system and pharmacological active substance system. The results are shown in **Figure 11** and **Figure 12**.

It can be seen from **Figure 11** that the NRBO-LSSVM-ABKDE model performs best among the four models, and it fitting degree (R=0.99998) is significantly higher than that of CPO-LSSVM (R=0.98974), PSO-LSSVM (R=0.88035) and NGO-LSSVM (R=0.90271). The distribution of predicted values and measured values is most concentrated near the reference line (Y=T), and the residual is the smallest, indicating that the model is highly accurate in modeling growth indicators.

**Figure 12** shows the prediction results of the secondary metabolite system. NRBO-LSSVM-ABKDE also showed the strongest predictive ability, and the fitting degree reached R=0.99999, which was significantly superior to other models (CPO-LSSVM: R=0.98899, PSO-LSSVM: R=0.88211, NGO-LSSVM: R=0.90695). Especially in the case of complex changes in the pharmacological active ingredients such as glycyrrhizic acid and liquiritin, the model can still accurately approximate the true value, reflecting its excellent modeling ability in complex nonlinear problems. Compared with the traditional optimization algorithm model, it is more suitable for the accurate prediction and simulation of *G.uralensis* under multivariate conditions.

#### 3.6.2 Analysis of model prediction results

**Figure 13:**
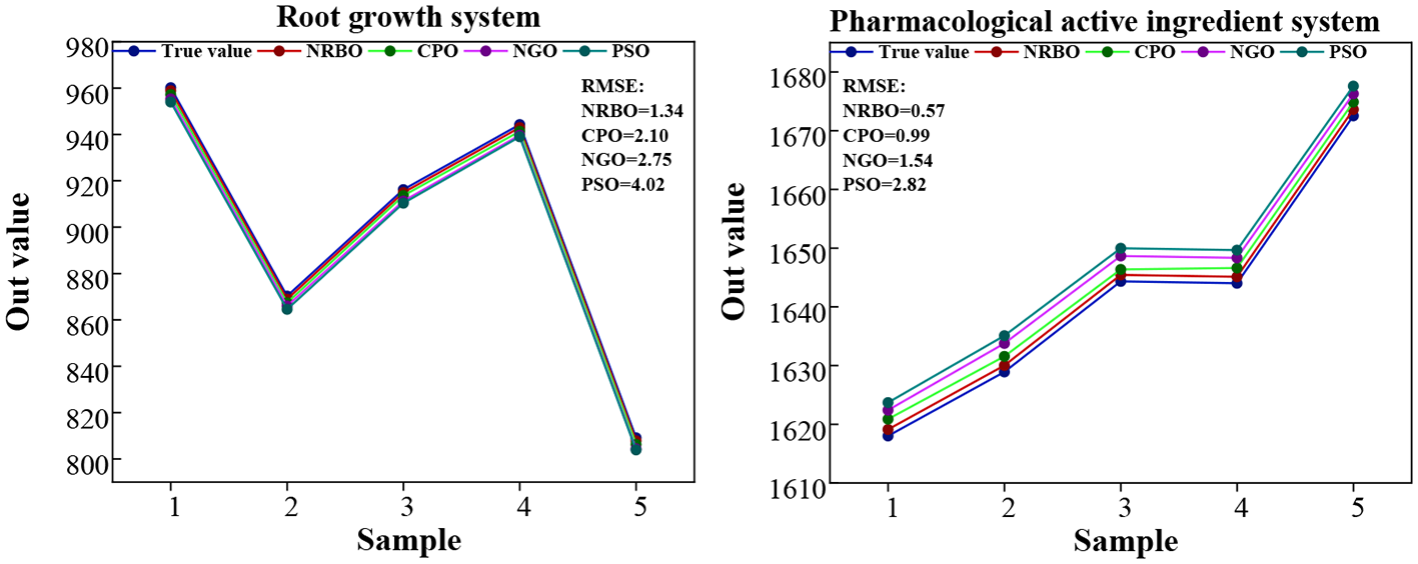
The prediction results of the four models in the S + La_0.75_ group for the two index systems of *G.uralensis*.

It can be seen from **Figure 13** that the prediction curve of NRBO-LSSVM-ABKDE model is the closest to the actual trend and the error is the smallest in both the growth system and the pharmacological active substance system prediction. Almost all of the five sample points fit the real change trajectory, showing excellent fitting ability. Compared with the other three models, the RMSE values of NRBO model were 1.34 and 0.57, respectively, which were significantly lower than those of CPO (2.10 and 0.99), NGO (2.75 and 1.54) and PSO (4.02 and 2.82), indicating that the model had stronger nonlinear fitting ability and was suitable for accurate modeling and prediction of complex physiological and growth data.

## 4 Discussion

### 4.1 Effects of different concentrations of La(NO_3_)_3_ on physiology and growth of G.uralensis under salt stress

#### 4.1.1 Effects of La(NO_3_)_3_ on the photosynthetic characteristics of G. uralensis under NaCl stress

Photosynthesis is an important process of plant metabolism, essential for growth and development (Ihsan et al., 2024). Net photosynthetic rate, stomatal conductance, intercellular CO_2_ concentration, and transpiration rate are core parameters for measuring photosynthetic efficiency of leaves. Salt stress, as a common environmental stress, inhibits these photosynthetic parameters in various ways, negatively impacting the normal photosynthetic functions of plants.

This study revealed that specific leaf area, net photosynthetic rate, stomatal conductance, intercellular CO_2_ concentration, and transpiration rate of *G. uralensis* significantly decreased under saline condition, corroborating the findings of Lopez-Climent (López-Climent et al., 2007) and Li (Li et al., 2024), which further confirm the negative effects of salt stress on plant photosynthesis. Notably, the application of an appropriate concentration of La(NO_3_)_3_ significantly improved these photosynthetic parameters, suggesting that lanthanum nitrate may alleviate the inhibitory effects of salt stress on photosynthesis via some mechanism.

At the molecular level, the application of La(NO_3_)_3_ appears to enhance photosynthetic capacity through a multi-faceted mechanism. Firstly, La³^+^ ions likely interact with key components of the photosynthetic apparatus. The suitable concentration of La(NO_3_)_3_ appears to delay chlorophyll degradation and maintain its stability, thereby prolonging its functional duration and enhancing the plant’s potential of light energy capture and utilization (Chen et al., 2024a). This stabilization may occur through the modulation of chlorophyllase and pheophytinase activities, enzymes responsible for chlorophyll breakdown, or by protecting chlorophyll molecules from salt-induced oxidative damage. Additionally, it may increase the number of chloroplasts, indicating a higher number of photosynthetically active units, which improves the plant’s photosynthetic capability (Janani et al., 2024). This increase could be linked to La³^+^-mediated upregulation of genes involved in chloroplast biogenesis, such as those encoding Golden-like proteins or other key transcription factors. Secondly, La(NO_3_)_3_ plays a crucial role in optimizing the structure and function of photosystems and the Calvin cycle. La(NO_3_)_3_ also enhances the structure of chloroplasts and induces the formation of the ribulose-1,5-bisphosphate carboxylase/oxygenase (Rubisco) complex and Rubisco activase, which enhances the activity of PSII protein complexes, improves photosynthetic electron transport efficiency, and increases CO_2_ assimilation efficiency, benefiting the development of photosystems I and II in chloroplasts (Franz et al., 2015; Ying and Juming, 2022). Specifically, La³^+^ ions may bind to the oxygen-evolving complex (OEC) of PSII, stabilizing its structure and preventing the disassembly of manganese clusters under salt stress, thereby maintaining high electron transport rates. Furthermore, the enhanced formation and activity of the Rubisco complex and Rubisco activase suggest that La³^+^ may influence the expression of rbcL and rbcS genes (encoding Rubisco subunits) and RCA genes, or directly modulate the activity of these enzymes through ionic interactions, leading to more efficient carbon fixation. Therefore, La(NO_3_)_3_ may improve the photosynthetic capacity of plants by enhancing the development of these two photosystems, ultimately promoting the growth and salt tolerance of *G. uralensis.*

#### 4.1.2 Effects of La(NO_3_)_3_ on antioxidant activity of G. uralensis under NaCl treatment

Osmotic stress and ion toxicity induced by salt stress can lead to the overproduction of reactive oxygen species (ROS) in plants (Faisal and Muhammad, 2021). ROS are oxygen-containing molecules with strong oxidative properties (Zhao et al., 2025). Under normal condition, their production and removal remain balanced. However, prolonged salt stress disrupts this balance, leading to excess ROS production and oxidative damage, which may induce membrane lipid peroxidation and damage cellular membranes, and even result in cell death (Aigerim et al., 2021). In response to elevated ROS levels, plants activate their antioxidant defense systems, increasing the activities of antioxidant enzymes such as SOD, CAT, and POD to mitigate the oxidative damage (Ghosh et al., 2024). It is demonstrated that the increased activities of these enzymes are closely linked to reduced ROS levels and enhanced salt tolerance of plants.

In our study, the activities of SOD, POD and CAT in leaves and roots of *G. uralensis* were significantly decreased under salt stress, while the content of MDA was significantly increased. This is consistent with the findings of Yu et al (Yu et al., 2019), which further explains that salt stress leads to a significant increase in ROS levels in silver mold, which in turn leads to membrane lipid peroxidation.

Application of an appropriate amount of lanthanum nitrate could effectively activate the licorice’s antioxidant defense system and reduce oxidative damage. It significantly enhanced the activities of SOD, POD, and CAT in both its leaves and roots, while notably reducing MDA content. These findings corroborate the general consensus established by previous studies, such as those by Tong et al. (2024) and Huang et al (Huang and Shan, 2018), which demonstrated that lanthanum application could enhance antioxidant enzyme activities and lower MDA content under various stress conditions.

However, a more critical comparison reveals both consistencies and distinctions. For instance, while Tong et al. reported a significant increase in SOD activity in maize under drought stress, the magnitude of the increase observed in our study with *G. uralensis* under salt stress was approximately 20% greater. This discrepancy may be attributed to the different stress types (drought vs. salinity) and/or species-specific responses to lanthanum. Salinity stress, involving both ionic toxicity and osmotic stress, might impose a more severe oxidative burden, thereby eliciting a stronger response from the antioxidant system when primed by La(NO_3_)_3_.

Furthermore, our study provides a more nuanced understanding by quantifying the response in both leaves and roots separately. The work of Huang et al. primarily focused on shoot responses, whereas our data indicate that the root system of *G. uralensis* exhibited a more pronounced relative increase in POD and CAT activities compared to the leaves. This suggests that La(NO_3_)_3_ might play a particularly crucial role in bolstering the antioxidant defense in the roots, the primary site of salt ion encounter, thereby protecting root integrity and function. This organ-specific response is a critical insight that extends the findings of previous research.

Therefore, while our results align with the broader principle that lanthanum mitigates stress-induced oxidative damage, they also highlight the context-dependent nature of this effect, emphasizing the roles of stressor type, plant species, and even the specific plant organ. Our findings thus not only confirm but also refine and expand upon the existing literature, providing a more comprehensive picture of lanthanum’s protective mechanisms.

#### 4.1.3 Effects of La(NO_3_)_3_ on root growth, biomass and its allocation of G. uralensis under NaCl stress

The effects of salt stress on plant root growth are complex. Root is a primary organ for water and mineral element absorption and is also highly sensitive to salt stress (Sharavdorj et al., 2024; Zhao et al., 2025). In high-salinity soil, the roots of *G. uralensis* mitigate the negative impacts of salt stress by regulating ion channel activity and producing antioxidant substances (Dilfuza et al., 2021; Xin et al., 2024). Our results demonstrated that NaCl stress significantly reduced the primary root length, root diameter, total root length, average root diameter, root volume, and the number of root branches of *G. uralensis*, confirming the inhibitory effects of salt stress on root development (Lang et al., 2020).

However, growth and development of root system was significantly improved by applying exogenous La(NO_3_)_3_. Increased activity of antioxidant enzymes and the regulation of osmotic adjustment substances in the roots by La(NO_3_)_3_ may be responsible for this improvement. Simultaneously, the reduced MDA content enhanced root activity, promoting root growth and improving the water and nutrient absorption capacity of the licorice (Xie et al., 2014).

NaCl stress also decreased biomass accumulation and the root-to-shoot ratio in *G. uralensis* (Dilfuza et al., 2016; Tong et al., 2019). However, exogenous La(NO_3_)_3_ application largely increased biomass and root-to-shoot ratios (Jia et al., 2024), suggesting that lanthanum may improve the adaptability and growth performance of *G. uralensis* by changing the allocation pattern of photosynthetic products in a saline environment. The molecular mechanism by which lanthanum alters nutrient allocation in *G. uralensis* requires further investigation.

#### 4.1.4 Effects of La(NO_3_)_3_ on medicinal secondary metabolites in G. uralensis under NaCl treatment

Secondary metabolites play a critical role in plant defense and adaptation to environmental stress. Although not directly involved in essential life processes, they contribute to the plant’s resilience to the adverse condition, provide protection, and facilitate interactions with other organisms (Bali et al., 2020). These compounds are particularly important in medicinal plants like *G. uralensis*, where they form the basis for their medicinal properties.

Under severe salt stress (160 mM NaCl), the content of secondary metabolites declined sharply, potentially reflecting an inhibition of the plant’s metabolic functions under stress condition. This result differs from the findings of Adinath et al. (2022) and Wang et al (Lanxiang et al., 2022), who found that salt stress significantly promoted the accumulation of flavonoid. The discrepancy may be due to the different responses of various plant species to salt stress. Notably, this study focused on changes in secondary metabolites in 1-year-old *G. uralensis* seedlings, and further research is required to understand the effects of salt on secondary metabolites in perennial individuals of the licorice.

In addition, we found that the application of La(NO_3_)_3_ under NaCl stress could significantly increase the content of medicinal substances in the roots of *G. uralensis*. This may be due to the fact that La(NO_3_)_3_ stimulates the expression of specific genes involved in the synthesis of these secondary metabolites, possibly by interfering with signal transduction, altering the accumulation of reactive oxygen species (Mengzhu et al., 2021; Dongwu et al., 2016; Xinyan et al., 2023).

### 4.2 Prediction of the mitigation effect of lanthanum nitrate on G.uralensis under salt stress

In this study, the NRBO-LSSVM-ABKDE coupling prediction model was innovatively introduced to systematically study the related indexes of *G.uralensis* under salt stress and exogenous lanthanum nitrate treatment. The experimental results show that the prediction results of the NRBO-LSSVM-ABKDE model are highly consistent with the trend of the measured data, especially under the input of multiple sets of variables, the model shows higher prediction accuracy and stability. Especially at 0.75 mM lanthanum concentration (S + La_0.75_), the model prediction effect is particularly significant. The stability and reliability of the model under multivariate input are proved.

In order to ensure the stability and reliability of the prediction model, this study further uses three other common optimization algorithm models (CPO-LSSVM, PSO-LSSVM, NGO-LSSVM) to compare with it. The results showed that the NRBO-LSSVM-ABKDE model showed the best performance in the prediction of the two systems by comparing the fitting effects of different models in the prediction of the growth system and the secondary metabolite system. Similarly, from the error analysis of the model, the NRBO-LSSVM-ABKDE model shows higher prediction accuracy in the classification data of growth and pharmacological activity, which further proves the strong nonlinear fitting ability of the NRBO-LSSVM-ABKDE model and its advantages in the modeling of complex physiological and growth data.

In summary, the NRBO-LSSVM-ABKDE coupled prediction model shows excellent performance in multi-task modeling such as growth and pharmacological active ingredient prediction of *G.uralensis*, and can accurately reflect the growth dynamics of *G.uralensis* and the accumulation trend of medicinal ingredients. This model can be integrated into farm management systems to provide actionable intelligence. For instance, farmers and cultivators could use the model’s real-time predictions to optimize irrigation schedules and fertilizer application rates, tailoring inputs to the precise physiological needs of the plants at different growth stages. This targeted approach not only maximizes resource use efficiency but also minimizes environmental runoff, promoting sustainable farming practices. Furthermore, for the pharmaceutical and herbal supplement industries, the model offers a robust method for dynamic quality evaluation. By predicting the optimal harvest time when the concentration of active ingredients (such as glycyrrhizin) peaks, producers can ensure batch-to-batch consistency and superior product quality, directly impacting economic value. Therefore, the NRBO-LSSVM-ABKDE model is not merely a predictive tool but a practical engine for driving the intelligent cultivation and high-quality production of licorice. Its implementation paves the way for data-driven decision-making in the cultivation of Chinese medicinal materials, ultimately fostering the sustainable and profitable development of the entire licorice industry.

## 5 Conclusion

This study focused on the effects of salt stress on the physiological characteristics and growth characteristics of *G.uralensis*, and the mitigation effect of exogenous lanthanum nitrate on salt stress, and carried out systematic experiments and modeling analysis. The results showed that salt stress significantly inhibited the photosynthesis and anti-oxidative stress ability of *G.uralensis*, resulting in plant growth retardation and decreased accumulation of medicinal components. The application of exogenous lanthanum nitrate effectively alleviated the adverse effects of salt stress, among which 0.75 mM concentration had the best effect, which showed the increase of photosynthetic efficiency and antioxidant enzyme activity, significantly improved the physiological state and growth performance of plants, and increased the accumulation of medicinal active ingredients in *G.uralensis*.

In order to further improve the accuracy and scientificity of the experiment, this study introduced the NRBO-LSSVM-ABKDE coupling prediction model, constructed the response system of *G.uralensis* under salt stress, and dynamically predicted the growth and pharmacological active components of *G.uralensis*. The model showed excellent fitting ability and stability under multivariate input, especially under the condition of 0.75 mM lanthanum nitrate treatment, the prediction effect was the best, which was better than other optimization algorithm models. It shows that the model can accurately reflect the physiological changes and component accumulation trend of *G.uralensis* under salt stress and lanthanum nitrate treatment, and has a good application prospect.

In summary, this study not only revealed the multi-dimensional influence mechanism of salt stress on *G.uralensis*, but also provided a scientific basis for lanthanum nitrate to alleviate salt damage, and provided an effective tool for precise cultivation and medicinal quality regulation of *G.uralensis*. However, it is important to acknowledge the limitations of our study. The findings are primarily derived from *G. uralensis* under specific salt stress conditions; therefore, the generalizability of the alleviating effects of lanthanum nitrate to other plant species or its efficacy under different types of abiotic stresses (e.g., drought, heavy metals) remains to be further investigated. These limitations, in fact, highlight promising avenues for future research. In the future, multi-omics technology can be employed to further dissect the lanthanum regulation mechanism across a broader range of species and stress conditions, which will not only validate the universality of our findings but also promote the sustainable development of the licorice industry and beyond.

## Data Availability

The raw data supporting the conclusions of this article will be made available by the authors, without undue reservation.
